# WWP2 protects against sepsis-induced cardiac injury through inhibiting cardiomyocyte ferroptosis

**DOI:** 10.2478/jtim-2024-0004

**Published:** 2024-03-21

**Authors:** Zhi Li, Boquan Wu, Jie Chen, Ning Ye, Rui Ma, Chunyu Song, Yingxian Sun, Xingang Zhang, Guozhe Sun

**Affiliations:** Department of Cardiology, The First Hospital of China Medical University, Shenyang, 110001, Liaoning Province, China

**Keywords:** WW domain-containing E3 ubiquitin protein ligase 2, sepsis, cardiac injury, oxidative stress, ferroptosis

## Abstract

**Background and Objectives:**

Cardiac injury plays a critical role in contributing to the mortality associated with sepsis, a condition marked by various forms of programmed cell deaths. Previous studies hinted at the WW domain-containing E3 ubiquitin protein ligase 2 (WWP2) involving in heart failure and endothelial injury. However, the precise implications of WWP2 in sepsis-induced cardiac injury, along with the underlying mechanisms, remain enigmatic.

**Methods:**

Sepsis induced cardiac injury were constructed by intraperitoneal injection of lipopolysaccharide. To discover the function of WWP2 during this process, we designed and performed loss/gain-of-function studies with cardiac-specific vectors and WWP2 knockout mice. Combination experiments were performed to investigate the relationship between WWP2 and downstream signaling in septic myocardium injury.

**Results:**

The protein level of WWP2 was downregulated in cardiomyocytes during sepsis. Cardiac-specific overexpression of WWP2 protected heart from sepsis induced mitochondrial oxidative stress, programmed cell death and cardiac injury, while knockdown or knockout of WWP2 exacerbated this process. The protective potency of WWP2 was predominantly linked to its ability to suppress cardiomyocyte ferroptosis rather than apoptosis. Mechanistically, our study revealed a direct interaction between WWP2 and acyl-CoA synthetase long-chain family member 4 (FACL4), through which WWP2 facilitated the ubiquitin-dependent degradation of FACL4. Notably, we observed a notable reduction in ferroptosis and cardiac injury within WWP2 knockout mice after FACL4 knockdown during sepsis.

**Conclusions:**

WWP2 assumes a critical role in safeguarding the heart against injury induced by sepsis via regulating FACL4 to inhibit LPS-induced cardiomyocytes ferroptosis.

## Introduction

Sepsis, a life-threatening syndrome caused by infection, impacts various organ systems in the body.^[1,2]^ Despite substantial global progress in promptly diagnosing and treating sepsis, the resulting dysfunction in multiple organs continues to be a significant contributor to mortality and morbidity.^[3,4]^ Among these dysfunctions, sepsis-induced myocardial dysfunction is prevalent, occurring at a rate of approximately 50%.^[5]^ Septic cardiomyopathy is marked by acute, extensive, and severe yet reversible heart dysfunction.^[6]^ This condition leads to inflammatory infiltration and structural alterations in the myocardium, often yielding unfavorable clinical outcomes.^[7]^ Currently, while factors like inflammation, oxidative stress, mitochondrial dysfunction, and programmed cell death have been suggested as playing a role in this process,^[8]^ the precise mechanisms behind the emergence and progression of sepsis-induced cardiac injury (SICI) remain incompletely comprehended.

Differing from apoptosis, necroptosis, and necrosis, ferroptosis stands as a novel form of controlled cellular demise, relying on ion and lipid peroxidation processes.^[9]^ Cells undergoing ferroptosis manifest distinct morphological and metabolic shifts, including mitochondrial atrophy, disruption of mitochondrial cristae, imbalanced lipid metabolism characterized by peroxidation of phospholipids containing polyunsaturated fatty acid (PUFA) chains, and accumulation of reactive oxygen species (ROS).^[10,11]^ Growing evidence points to the involvement of ferroptosis in the progression of diverse cardiovascular disorders. For example, the utilization of ferroptosis inhibitors demonstrates a capacity to diminish myocardial ischemia-reperfusion (IR) injury and enhance cardiac performance.^[12]^ In instance of doxorubicin-induced cardiotoxicity, the reduction of glutathione peroxidase 4 (GPX4) results in heightened lipid oxidation and initiates ferroptosis, whereas the elevation of GPX4 suppresses ferroptosis, thereby mitigating doxorubicin-induced myocardial harm and metabolic disturbances.^[13]^ Up to the present time, preliminary investigations propose that ferritinophagy-mediated ferroptosis plays a role in sepsis-induced cardiac damage.^[14]^ Nonetheless, the mechanisms through which LPS (lipopolysaccharide)-induced burst of ROS and disruption of mitochondrial metabolism trigger ferroptosis in cardiac cells, along with their potential pathways, remain shrouded in mystery.

The ubiquitin-proteasome system, an essential cellular mechanism responsible for regulating protein balance, plays a significant role in both maintaining and influencing various cardiovascular pathophysiological processes.^[15,16]^ WW domain-containing E3 ubiquitin protein ligase 2 (WWP2), an E3 ubiquitin ligase falling under the C2-WW-HECT category, contributes to several intracellular biological processes by overseeing the degradation of multiple substrate proteins.^[17,18]^ Previous research indicated that WWP2 was connected to diverse pathological mechanisms, including tumorigenesis, oxidative stress, and the remodeling of ventricular and endothelial cells.^[19]^ This connection is established through its influence on cell differentiation, immune response, and intracellular signaling.^[20,21]^ Nonetheless, the extent of WWP2’s involvement in mitochondrial ROS (reactive oxygen species)-dependent ferroptosis within cardiac cells and its role in LPS-induced cardiac injury remains uncertain.

In this study, we uncovered that LPS has the ability to trigger multiple forms of programmed cell death within cardiac cells, with ferroptosis being the most characteristic. Moreover, our findings point to the involvement of WWP2 in mediating LPS-induced cardiac injury. Through the utilization of WWP2 knockout (WWP2KO) mice and the deployment of cardiac cell-specific WWP2 intervention viral vectors, we found that WWP2 predominantly ameliorates LPS-induced cardiac injury and bolsters cardiac function. This protection is achieved by curbing the generation of mitochondrial ROS and suppressing ferroptosis induced by lipid peroxidation. The underlying mechanism hinges on WWP2’s ability to facilitate the degradation of FACL4 (long chain fatty acid-CoA ligase 4), a metabolic enzyme integral to the synthesis of PUFAs (polyunsaturated fatty acids), using the ubiquitin-proteasome system. This degradation process effectively counteracts disruptions in intracellular lipid metabolism and the initiation of iron-dependent cell death prompted by LPS, thereby bestowing protective effects upon the heart in the face of LPS-induced injury.

## Materials and methods

### Animals

Animal experiments in this study were approved by the Animal Subject Committee of China Medical University (CMU2023364). Wide type (WT) male C57BL/6J mice were purchased from the animal center of China Medical University. WWP2KO mice were constructed by the Shanghai Model Organisms Center, Inc (Shanghai, China) and were confirmed by polymerase chain reaction (PCR) using primers as follows: Forward: 5’-TGGGCGTGTCTATTATGTTGAT-3’; Reverse: 5’-GTTGTGGTCCACGTAGTAAAAC-3’.

### Animal Studies

Mice were reared in a SPF (specific pathogen free) environment under a 12 h light/dark cycle. 8-week-old C57BL/6J male mice were used for the following experiments: Mouse model of sepsis was established by intraperitoneally injection with LPS (10 mg/kg) and mice injected with saline were served as controls. Six hours after the LPS injection, mice were intraperitoneally injected with Fer-1 (1 mg/kg) to inhibit ferroptosis. For *in vivo* intervention, WT mice were injected with AAV9-cTNT-Flag-WWP2, AAV9-cTNT-WWP2sh, or AAV9-cTNT-FACL4sh respectively 14 days before the LPS treatment through tail vein (0.1 mL; 5 × 10^12^ PFU/mL). AAV9-cTNT-Flag-CON or AAV9-cTNT-CONi was used as a empty vector.

### Western Blot

Myocardial tissue or cell samples were lysed in lysis buffer containing 1% protease inhibitors. Protein complex was then collected by centrifugation at 12, 000 rpm for 60 min at 4°C. Prepared protein samples were separated by SDS-PAGE (Sodium Dodecyl Sulfate-Polyacrylamide Gel Electrophoresis) gels and the transferred to PDVF (Polyvinylidene fluoride) membranes. The membranes were blocked with 5% non-fat milk, followed by incubation with primary antibodies at 4°C overnight and secondary antibodies at room temperature for 2 h. The protein bands were visualized by chemiluminescence (Tanon Science& Technology Co., Ltd., Shanghai, China) and quantified using ImageJ software version 1.46 (National Institutes of Health, USA). The primary antibodies used in this study are as follows: α-TUBULIN (Proteintech, 11224–1-AP, diluted 1: 1000), WWP2 (Proteintech, 12197–1-AP, diluted 1: 1000), GPX4 (Proteintech, 67763–1-Ig, diluted 1: 1000), FACL4 (Proteintech, 22401–1-AP, diluted 1: 1000), PTGS2 (Proteintech, 66351–1-Ig, diluted 1: 1000), Flag (Proteintech, 80010-1-RR, diluted 1: 1000).

### Immunofluorescence Assays

Paraffin-fixed myocardium slices were used for deparaffinization and antigen retrieval. After washed with PBS (phosphate belanced solution) for three times, the slices were blocked and permeabilized with 10% BSA (Bovine Serum Albumin) plus 0.1% Triton X-100 in PBS for 2 h. The slices were then incubated with primary antibodies overnight at 4°C and secondary antibodies for another 2 h. The nucleus was stained with 4’, 6-diamidino-2-phenylindole (DAPI; C0065, Solarbio, Beijing, China).

TUNEL (Terminal deoxynucleotidyl transferase-mediated dUTP nick end labeling) staining was performed with the TUNEL Apoptosis Detection Kit (KTA2011, Abbkine, USA) following the manufacturer’s instructions. ROS detection was conducted in frozen sections of fresh myocardium incubated with the Dihydroethidium (DHE) probe for 30 min at room temperature.

For cell immunofluorescent staining, H9C2 cells were fixed with 4% paraformaldehyde for 15 min, followed by blocked with 10% BSA and incubation with primary and secondary antibodies.

The primary antibodies used in this study are as follows: WWP2 (Proteintech, 12197–1-AP), cTNT (Proteintech, 68300–1-Ig), CD31 (Proteintech, 66065–2-Ig), α-SMA (Proteintech, 67735–1-Ig), 4-HNE (Invitrogen, MA5–27570), FACL4 (Proteintech, 22401–1-AP). The images were observed and taken under Nikon A1 confocal microscope.

### Histological Analysis

Paraffin-fixed myocardium tissue were sliced into 5 μm sections. The hematoxylin and eosin (H&E) staining was performed according to the standard procedures. For immunohistochemistry staining, myocardial slices were blocked with 10% BSA, followed by incubation with primary antibodies against WWP2 (diluted 1: 100) overnight at 4°C. Slices were then incubated with secondary antibodies and the images were taken under a microscope.

### Cell Culture and Interventions

H9C2 cells were cultured in Dulbecco’s modified Eagle’s medium (DMEM, HyClone, Logan, UT, USA) with 10% fetal bovine serum (FBS, HyClone, Logan, UT, USA) at 37°C in a humidified atmosphere with 5% CO_2_. For WWP2 or FACL4 intervention, plasmids transfection was performed with Lipofectamine 3000 (Invitrogen, California, USA), according to the manufacturer’s instructions for 48 h. Knockdown of WWP2 or FACL4 was performed with siRNAs and verified by Western blot. For *in vitro* models of sepsis, LPS was added in culture medium for 6 h. The target gene intervention was performed 6 h before LPS treatment. Mitochondrial membrane potential in H9C2 cells were detected with the JC-1 kit (Solarbio, M8650, China). In brief, the cells were incubated with culture medium for 20 min at 37°C and subsequent JC-1 staining working solution. The images were taken under a microscope. The detection of ROS content in H9C2 cells was performed with the MitoSOX Red (MCE, 1003197–00–9, USA) according to the protocols. The levels of lipid peroxidation products in H9C2 cells were visualized through the MitoPeDPP staining kit (DOJINDO, M466, Japan).

### Echocardiography

Cardiac function was evaluated by echocardiography. Briefly, mice were anesthetized with 1.5% isoflurane. Two-dimensional M-mode recording was used to assess cardiac function on a Visual Sonics Vevo 2100 real-time, high-resolution *in vivo* micro-imaging system (Visualsonic, Canada). The left ventricular ejection fraction (LVEF), left ventricular fractional shortening (LVFS), interventricular septal thickness at diastole (IVSd), left ventricular posterior wall dimensions (LVPWd) and left ventricular end diastolic (LVd) were measured and quantified.

### Myocardial Injury and Inflammation Markers Testing

The levels of myocardial injury and inflammation factors (LDH (Lactate Dehydrogenase), CK-MB (creatine kinase-MB), cTNI (cardiac troponin I) and TNF-α (tumor necrosis factor-α)) were detected using assay kits (Solarbio, BC0685, SEKM-0152, SEKM-0153, SEKM-0034, China) under the manufacturer’ s instruction.

### Transmission Electron Microscopy (TEM)

Fresh heart pieces (< 1 mm^3^) were fixed with electron microscope fixation solution at 4°C for 2 h, followed by fixation with 1% osmic acid at room temperature for 2 h. The heart sections were then embedded in Epon-Araldite resin. Images were observed and acquired with Transmission electron microscopy (HITACHI, HT7700, Japan).

### Malondialdehyde (MDA) Detection

Myocardial lipid peroxidation was analyzed by quantification of MDA content with the MDA Content Assay Kit (Solarbio, BC0025) under the instruction. Briefly, mice myocardium was lysed at 4°C. After centrifuged at 8000 g for 10 min, the supernatant was collected for absorbance measurement with a spectrophotometer.

### Measurement of GSH (Glutathione) and GSSG (glutathione disulfide)

The GSH and GSSG content was detected with the GSH and GSSG Assay Kit (Beyotime, S0053) according to the manufacturer’s instructions.

### Co-immunoprecipitation and Ubiquitylation Assays

H9C2 cells or myocardium was lysed on ice for 1 h after treatment. The supernatant was collected after centrifugation at 12, 000 rpm for 40 min at 4°C and incubated with anti-WWP2 or anti-FACL4 antibodies for 6 h, followed by incubation with protein A/G agarose beads (Santa Cruz Biotechnology, USA) for another 12 h at 4°C. The protein complex was then detected by WB (Western blot). For ubiquitylation assays, FACL4 ubiquitination was analyzed by WB with anti-Ub antibody (Proteintech, 10201–2-AP).

### Statistical Analysis

All statistical analysis were performed with Prism 8 (GraphPad, La Jolla, CA) and all quantitative data were presented as the mean±SEM. Comparison between two groups was performed with unpaired Student t-tests and statistical differences among > 2 groups were analyzed with one-way ANOVA with Dunnett’s multiple comparison post-hoc test or Tukey’s multiple comparison post-hoc test. Statistical significance was considered as *P* value < 0.05.

## Results

### WWP2 Was Downregulated in Septic Heart and Cardiomyocytes

**Figure 1:**
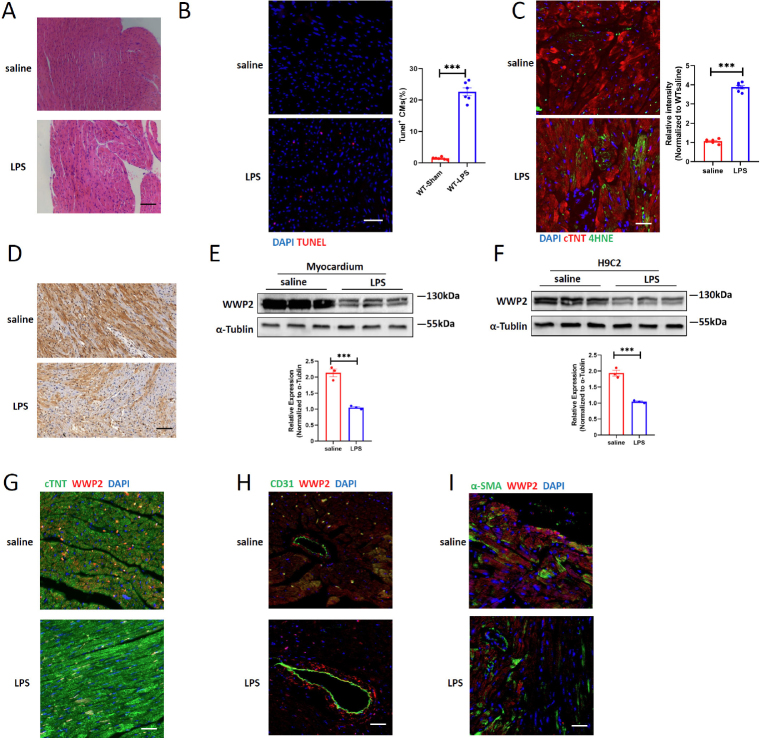
WWP2 is downregulated in septic heart and cardiomyocytes. (A) Representative H& E staining of myocardium in adult mice treated with saline or LPS. Scale bar: 50 μm. (B) Representative immunofluorescence (IF) staining and quantification of Tunel^+^ cardiomyocytes in myocardium treated with saline or LPS. Scale bar: 100 μm (*N* = 6). (C) Representative immunofluorescence staining and quantification of 4-HNE in myocardium treated with saline or LPS. Scale bar: 75 μm (*N* = 6). (D) Representative immunohistochemistry (IHC) staining of WWP2 in myocardium treated with saline or LPS. Scale bar: 75 μm. (E) Western blotting and quantification of WWP2 in in adult mice treated with saline or LPS (*N* = 3). (F) Western blotting and quantification of WWP2 in in H9C2 cell line treated with saline or LPS (*N* = 3). (G) Representative immunofluorescence (IF) staining of WWP2 and cardiomyocytes (cTNT) in myocardium treated with saline or LPS. Scale bar: 50 μm. (H) Representative immunofluorescence (IF) staining of WWP2 and Endothelial cells (CD31) in myocardium treated with saline or LPS. Scale bar: 50 μm. (I) Representative immunofluorescence (IF) staining of WWP2 and fibroblasts (α-SMA) in myocardium treated with saline or LPS. Scale bar: 50 μm. Data were presented as the mean±SEM. ^***^P<0.001.

To explore the role of WWP2 in SICI, we first examined the expression pattern of WWP2 in septic myocardium. For this purpose, we established an *in vivo* sepsis injury model through intraperitoneal injection of LPS, and a corresponding *in vitro* model utilizing LPS treatment on the H9C2 cell line. Employing H& E staining, we uncovered distinct features such as myofibril dissolution, interstitial edema, and inflammatory infiltration in sepsis-triggered myocardium (Figure 1A). TUNEL staining, alongside 4HNE (4-Hydroxynonenal) staining targeting lipid peroxidation products, collectively revealed that sepsis orchestrated programmed cardiomyocyte cell death, encompassing both apoptosis and ferroptosis (Figure 1B-C).

We further investigated the expression level of WWP2 in cardiac tissues treated with LPS. Immunohistochemistry results showed a decrease in WWP2 levels in cardiac tissues treated by LPS (Figure 1D). These findings were further substantiated by Western blotting analysis, which exhibited reduced WWP2 protein levels in both cardiac tissues and cardiomyocytes when compared to the control group after the introduction of LPS (Figure 1E-F). Given the broad expression of WWP2 across various cell types, we undertook co-immunofluorescence staining, pairing WWP2 with markers specific to cardiomyocytes, vascular endothelial cells, and fibroblasts. The results shed light on a marked reduction of WWP2 within cardiomyocytes following LPS treatment, while the expression levels in fibroblasts and endothelial cells remained relatively unchanged (Figure 1G-I). In summary, our observations collectively pointed to the downregulation of WWP2 within cardiomyocytes affected by sepsis.

### Knockdown of WWP2 Aggravates Myocardial Damage in Sepsis Models

**Figure 2:**
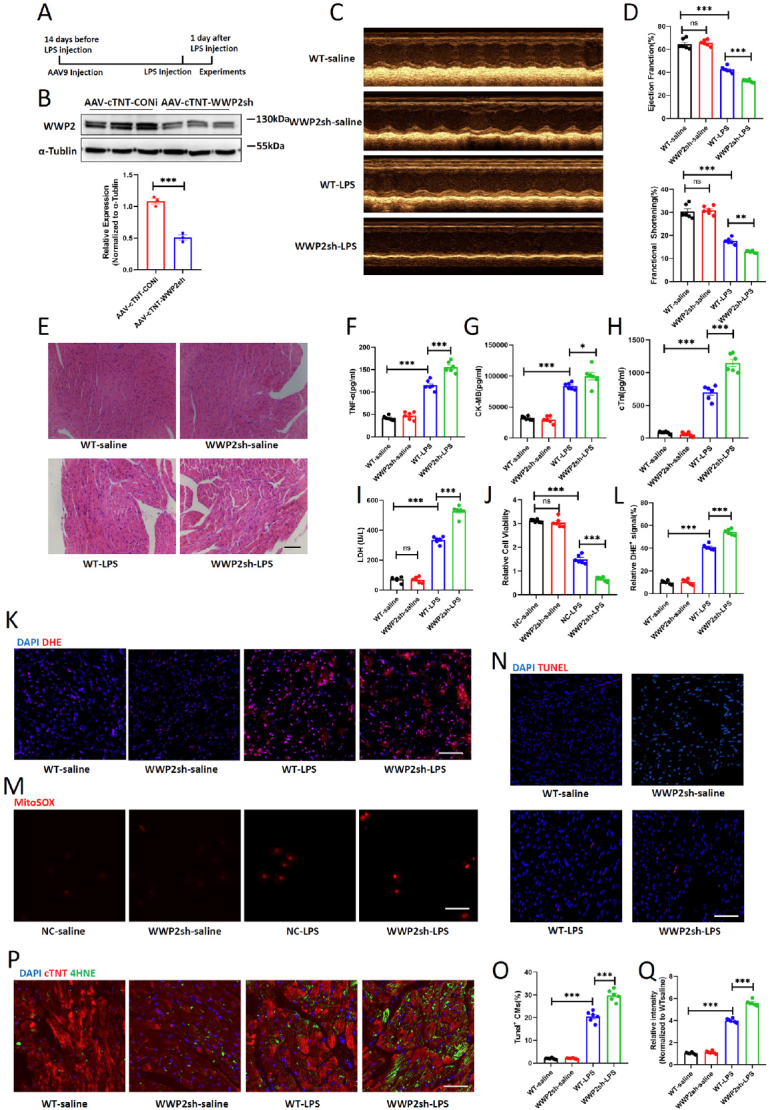
Knockdown of WWP2 aggravates myocardial damage in sepsis models. (A) Schematic illustration of the study design. (B) Western blotting and quantification of WWP2 in mice injected with AAV9-CTNT-WWP2sh or AAV9-CTNT-CONi. (*N* = 3). (C-D) Representative images of echocardiogram and quantification of ejection fraction (EF) and fractional shortening (FS) in WT-saline, WWP2sh-saline, WT-LPS and WWP2sh-LPS mice (*N* = 6). (E) Representative H& E staining of myocardium in WT-saline, WWP2sh-saline, WT-LPS and WWP2sh-LPS mice. Scale bar: 50 μm. (F-H) Detection of plasma levels of CK-MB, cTnI and TNF-α in WT-saline, WWP2sh-saline, WT-LPS and WWP2sh-LPS mice (*N* = 6). (I) Detection of LDH levels in WT-saline, WWP2sh-saline, WT-LPS and WWP2sh-LPS myocardium (N=6). (J) Cell viability quantified among NC-saline, WWP2sh-saline, NC-LPS and WWP2sh-LPS H9C2 cells by CCK-8 assay (*N* = 6). (K-L) Representative IF staining and quantification of DHE in WT-saline, WWP2sh-saline, WT-LPS and WWP2sh-LPS myocardium. Scale bar: 75 μm (*N* = 6). (M) Representative IF staining of mitoSOX in H9C2 cells treated with saline or LPS and transfected with WWP2 siRNA or empty vectors. Scale bar: 50 μm. (N-O) Representative IF staining and quantification of Tunel^+^ cardiomyocytes in WT-saline, WWP2sh-saline, WT-LPS and WWP2sh-LPS myocardium. Scale bar: 50 μm (*N* = 6). (P-Q) Representative IF staining and quantification of 4-HNE in WT-saline, WWP2sh-saline, WT-LPS and WWP2sh-LPS myocardium. Scale bar: 75 μm (*N* = 6). Data were presented as the mean±SEM. NS, not significant, ^*^P<0.05, ^**^P<0.01, ^***^P<0.001.

The reduced expression level of WWP2 in the LPS-induced cardiac injury model raises intriguing possibilities about its potential involvement. To meticulously examine the influence of WWP2 on sepsis-induced cardiac injury, we initiated our investigation by performing tail vein injections of AAV-CTNT-WWP2sh. Following a 14-day period, cardiac injury was induced by intraperitoneal injection of LPS (Figure 2A). The efficacy of myocardial WWP2 knockdown was confirmed through Western blot (Figure 2B). One day after injection, we employed echocardiography to assess mouse cardiac contractile function. The results unveiled that under normal physiological conditions, WWP2 knockdown didn’t impart significant effects on heart function. However, within the LPS-induced model, cardiac contractile function significantly deteriorated due to WWP2 knockdown, without any marked impact on indicators like ventricular wall thickness (Figure 2C-D, supplemental Figure 1). By performing H& E staining, we observed the disordered myocardium and inflammatory infiltration was further aggravated with WWP2 knockdown (Figure 2E). Furthermore, we found that WWP2 knockdown exacerbated the extent of injury. This was evident through noteworthy elevations in serum markers including CK-MB, cTNI, and TNF-α (Figure 2F-H). Additionally, the level of myocardial LDH (Lactate Dehydrogenase) was exacerbated by WWP2 knockdown (Figure 2I). Similar investigations were conducted in H9C2 cells, revealing that cell viability decreased due to LPS treatment and was further reduced after WWP2 knockdown (Figure 2J). Subsequently, we assessed the impact of WWP2 on oxidative stress injury in cardiac tissue. DHE staining results indicated that LPS induced cardiac oxidative stress injury, which was exacerbated by WWP2 knockdown (Figure 2K-L), which was in line with the mitochondrial ROS detection in H9C2 cells (Figure 2M). To explore the influence of WWP2 knockdown on programmed cardiac cell death, we utilized TUNEL staining and 4HNE staining (Figure 2N-Q). Our findings demonstrated that WWP2 knockdown aggravated LPS-induced cardiac cell apoptosis and ferroptosis.

### Cardiac-specific Overexpression of WWP2 Alleviates Sepsis-induced Myocardial Injury

**Figure 3:**
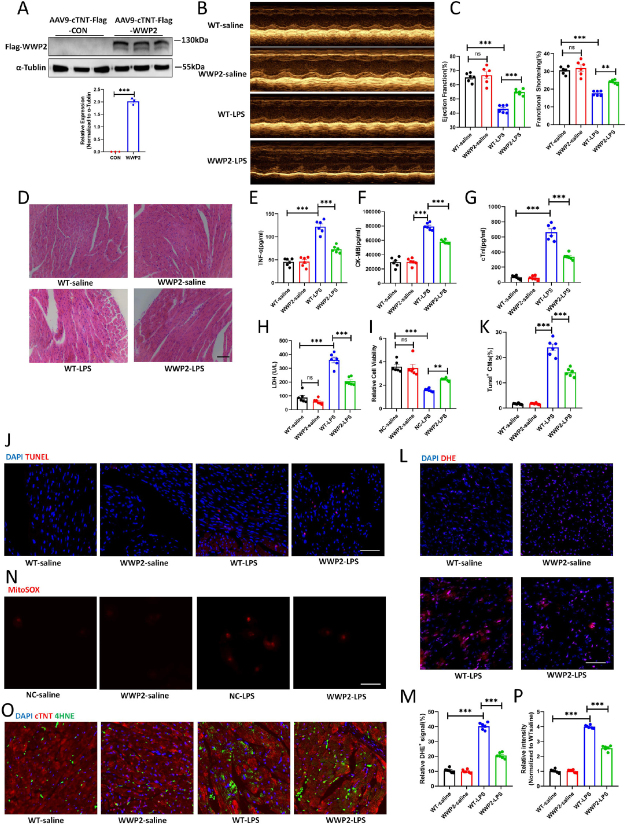
Cardiac-specific overexpression of WWP2 alleviates sepsis-induced myocardial injury. (A) Western blotting and quantification of WWP2 in mice injected with AAV9-CTNT-Flag-WWP2 or AAV9-CTNT-Flag-CON (*N* = 3). (B-C) Representative images of echocardiogram and quantification of ejection fraction (EF) and fractional shortening (FS) in WT-saline, WWP2-saline, WT-LPS and WWP2-LPS mice (*N* = 6). (D) Representative H& E staining of myocardium in WT-saline, WWP2-saline, WT-LPS and WWP2-LPS mice. Scale bar: 50 μm. (E-G) Detection of plasma levels of CK-MB, cTnI and TNF-α in WT-saline, WWP2-saline, WT-LPS and WWP2-LPS mice (*N* = 6). (H) Detection of LDH levels in WT-saline, WWP2-saline, WT-LPS and WWP2-LPS myocardium (*N* = 6). (I) Cell viability quantified among NC-saline, WWP2-saline, NC-LPS and WWP2-LPS H9C2 cells by CCK-8 assay (*N* = 6). (J-K) Representative IF staining and quantification of Tunel^+^ cardiomyocytes in WT-saline, WWP2-saline, WT-LPS and WWP2-LPS myocardium. Scale bar: 50 μm (*N* = 6). (L-M) Representative IF staining and quantification of DHE in WT-saline, WWP2-saline, WT-LPS and WWP2-LPS myocardium. Scale bar: 75 μm (*N* = 6). (N) Representative IF staining of mitoSOX in H9C2 cells treated with saline or LPS and transfected with WWP2 or empty plasmids. Scale bar: 50 μm. (O-P) Representative IF staining and quantification of 4-HNE in WT-saline, WWP2-saline, WT-LPS and WWP2-LPS myocardium (*N* = 6). Scale bar: 75 μm. Data were presented as the mean±SEM. NS, not significant, ^**^P<0.01, ^***^P<0.001.

To gain further insight into the distinct role of cardiac-specific WWP2, we executed tail vein injections of AAV-CTNT-Flag-WWP2, succeeded by intraperitoneal LPS injections 14 days later to establish the sepsis model. We first verified the successful overexpression of cardiac-specific WWP2 (Figure 3A). Following this, a series of assessments including cardiac ultrasound, H&E staining, and serum marker analyses were conducted. These investigations collectively demonstrated that WWP2 overexpression had a significant positive impact on SICI. It notably enhanced cardiac contractile function while mitigating the myocardial injury induced by LPS (Figure 3B-H, Supplemental Figure 2). *In vitro*, we also found that WWP2 significantly improved cardiomyocytes survival (Figure 3I). Additionally, we delved into the realms of myocardial oxidative stress and cell death. Intriguingly, WWP2 exhibited a notable capacity to suppress these detrimental processes triggered by sepsis-induced conditions (Figure 3J-P). These results collectively illustrate that WWP2 is a critical participant in sepsis-induced cardiac injury, and its overexpression can ameliorate LPS-induced cardiac injury.

### WWP2 Is Predominantly Responsible for Reducing ferroptosIs in sepsIs-induced Myocardial Damage

**Figure 4:**
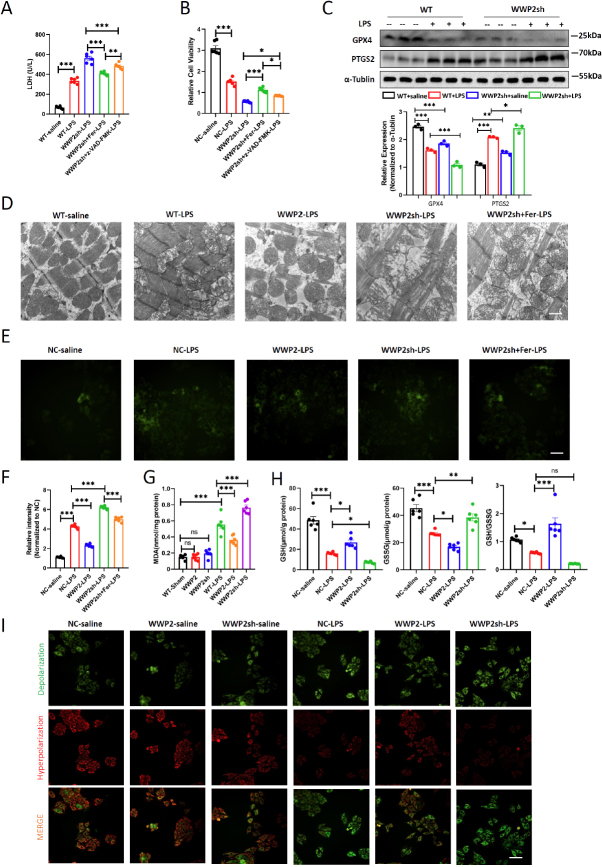
WWP2 is predominantly responsible for reducing ferroptosis in sepsis-induced myocardial damage. (A) Detection of LDH levels in NC-saline, NC-LPS, WWP2sh-LPS, WWP2sh+Fer-1-LPS and WWP2sh+z-VAD-FMK-LPS myocardium (*N* = 6). (B) Cell viability quantified among NC-saline, NC-LPS, WWP2sh-LPS, WWP2sh+Fer-1-LPS and WWP2sh+z-VAD-FMK-LPS H9C2 cells by CCK-8 assay (*N* = 6). (C) Western blotting and quantification of GPX4 and PTGS2 in mice treated with LPS or saline and injected with AAV9-CTNT-WWP2sh or AAV9-CTNT-CONi (*N* = 3). (D) Representative transmission electron microscopy (TEM) images of mitochondrial morphology and function. Scale bar: 800 nm. (E-F) Representative images and quantification of lipid peroxidation products in H9C2 cells. Scale bar: 20 μm (*N* = 6). (G) Determination of Malondialdehyde (MDA) levels in mice treated with LPS or saline and injected with AAV9-CTNT-WWP2, AAV9-CTNT-WWP2sh or AAV9-CTNT-CON (*N* = 6). (H) Determination of glutathione (GSH), Oxidized glutathione (GSSG) and GSH/GSSG ratio in H9C2 cells (*N* = 6). (I) Representative images of mitochondrial membrane potential stained by JC-1 kit. Scale bar: 50 μm. Data were presented as the mean±SEM. ^*^P<0.05, ^**^P<0.01, ^***^P<0.001.

To elucidate the microscopic intricacies of how WWP2 improves LPS-induced myocardial injury, we delved into the impact of WWP2 on two types of programmed cell death: apoptosis and ferroptosis. Employing tail vein injections of AAV9-CTNT-WWP2sh, we initiated the sepsis model after a 14-day period, simultaneously introducing either the apoptosis inhibitor z-VAD-FMK or the ferroptosis inhibitor Fer-1 through intraperitoneal injections. Our findings unveiled that, in comparison to z-VAD-FMK, Fer-1—known for its ferroptosis inhibiting properties— significantly ameliorated LDH levels exacerbated by WWP2 knockdown (Figure 4A). The impaired cardiomyocytes viability was also recovered after ferroptosis inhibition (Figure 4B). This observation leads us to believe that, while both apoptosis and ferroptosis are influenced by WWP2 in the regulation of LPS-induced myocardial injury, ferroptosis might hold a more prominent role. Therefore, we dug deeper into WWP2’s influence on cardiomyocytes ferroptosis within the sepsis cardiac injury model. Central to ferroptosis regulation is glutathione peroxidase 4 (GPX4) as a key inhibitory factor, and PTGS2 serves as a significant marker during this process. ^[22]^ Our Western blot results demonstrated that LPS reduced GPX4 levels while elevating PTGS2 levels, and WWP2 knockdown prompted this trend, suggesting that WWP2 knockdown induces cellular ferroptosis (Figure 4C). Drawing from extensive prior research suggesting that mitochondrial dysfunction and altered morphology are pivotal in cellular ferroptosis,^[23,24]^ we speculated that WWP2 could potentially modulate ferroptosis by influencing cardiac cell mitochondrial structure and function. To probe this hypothesis, we turned to transmission electron microscopy (TEM) and observed that LPS-induced damage was evident in cardiac tissue mitochondrial morphology. Strikingly, post-WWP2 knockdown, features like mitochondrial shrinkage, membrane disruption, and reduced cristae were more pronounced. In contrast, WWP2 overexpression displayed a distinct capability to ameliorate mitochondrial damage (Figure 4D). Subsequently, we found that WWP2 accelerated the clearance of lipid peroxidation products and MDA levels in cells treated with LPS. Moreover, shifts in oxidative-reductive balance were scrutinized (Figure 4E-G). LPS-induced myocardial injury resulted in decreased cardiac tissue GSH content and increased GSSG levels. Remarkably, WWP2 intervened to significantly improve this worsening trend, restoring oxidative-reductive balance (Figure 4H). The intricate realm of mitochondrial dynamics was also explored. LPS treatment was associated with a considerable reduction in mitochondrial membrane potential, a change that WWP2 was able to counteract, thereby maintaining mitochondrial stability (Figure 4I). In synthesis, our findings converge to suggest that WWP2’s primary mode of regulation in LPS-induced myocardial injury is linked to ferroptosis. It becomes apparent that WWP2 holds the capability to modulate aberrant mitochondrial morphology and function within the context of ferroptosis occurrence.

### WWP2 Promotes Ubiquitin-proteasome Pathway Dependent Degradation of FACL4

**Figure 5:**
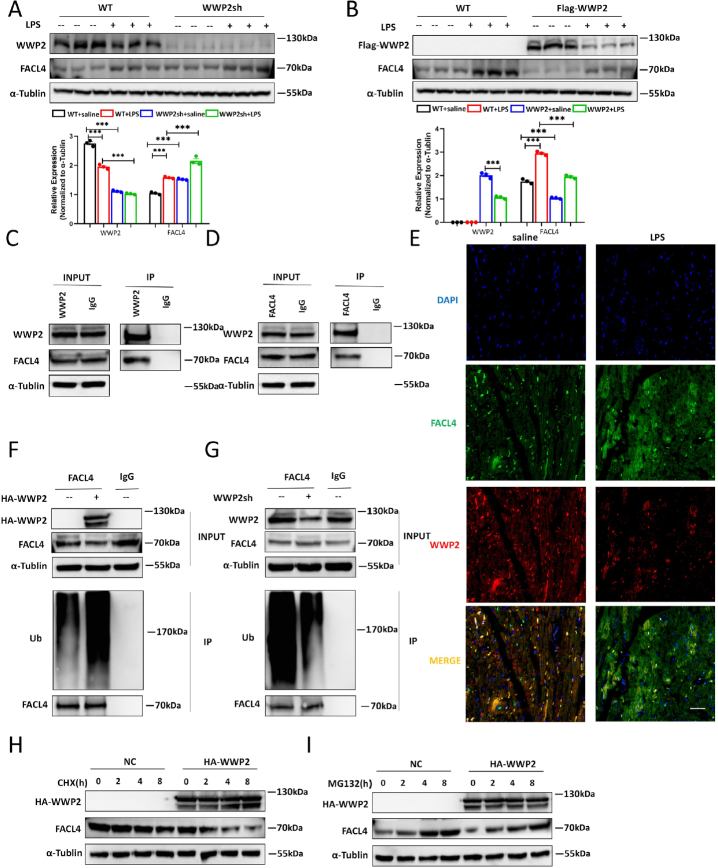
WWP2 promotes ubiquitin-proteasome pathway dependent degradation of FACL4. (A) Western blotting ang quantification of WWP2 and FACL4 in mice treated with LPS or saline and injected with AAV9-CTNT-WWP2sh or AAV9-CTNT-CONi (*N* = 3). (B) Western blotting and quantification of WWP2 and FACL4 in mice treated with LPS or saline and injected with AAV9-CTNT-Flag-WWP2 or AAV9-CTNT-Flag-CON (*N* = 3). (C) Coimmunoprecipitation and Western blotting analysis of FACL4 in 293T cells precipitated with anti-WWP2. (D) Coimmunoprecipitation and Western blotting analysis of WWP2 in 293T cells precipitated with anti-FACL4. (E) Representative images of co-localization of WWP2 and FACL4 in myocardium treated with saline or LPS. Scale bar: 50 μm. (F) Detection of ubiquitination levels in 293T cells transfected with HA-WWP2 plasmids. G. Detection of ubiquitination levels in 293T cells transfected with WWP2 shRNA. (H) Western blotting analysis of WWP2 and FACL4 in H9C2 cells treated with CHX and HA-WWP2. (I) Western blotting analysis of WWP2 and FACL4 in H9C2 cells treated with MG132 and HA-WWP2. Data were presented as the mean±SEM. ^***^P<0.001.

Continuing our pursuit of understanding the molecular underpinnings behind WWP2’s regulation of sepsis-induced myocardial injury and cardiomyocytes ferroptosis, our focus turned to the pivotal enzyme acyl-CoA synthetase long-chain family member 4 (FACL4). This enzyme is integral in the esterification process of arachidonic acid (AA) and adrenoyl into phosphatidylethanolamine (PE). This process is known to activate polyunsaturated fatty acids (PUFAs) and maintain organelle membrane homeostasis, a facet closely tied to ferroptosis.^[25]^ Given that we’ve already suggested WWP2’s involvement in regulating mitochondrial homeostasis-related ferroptosis in the previous results, we hypothesized that FACL4 might be a downstream target through which WWP2 exerts its effects. To investigate this, we treated H9C2 cells with LPS and manipulated WWP2 levels. The results from Western blot showed that FACL4 protein levels increased after LPS treatment. Overexpressing WWP2 reduced FACL4 protein levels in both control and LPS-treated groups, while WWP2 knockdown yielded the opposite results (Figure 5A-B). As WWP2 is a C2-WW-HECT-type E3 ubiquitin ligase, we further speculated that WWP2 might regulate FACL4 through the ubiquitin-proteasome pathway. Immuno-coprecipitation experiments revealed a direct interaction between WWP2 and FACL4 (Figure 5C-D), which was further verified by the immunofluorescent colocalization of WWP2 and FACL4 (Figure 5E). Furthermore, WWP2 overexpression elevated FACL4 ubiquitination levels, while WWP2 knockdown reduced it (Figure 5F-G). Importantly, this regulatory relationship was reversed when cells were treated with MG-132, a proteasome inhibitor (Figure 5H-I). Based on these findings, it’s plausible that WWP2 regulates cardiomyocytes ferroptosis levels by promoting the ubiquitination and subsequent degradation of FACL4.

### LPS-induced Myocardial Injury and FerroptosIs Is Exacerbated in WWP2KO Mice

**Figure 6:**
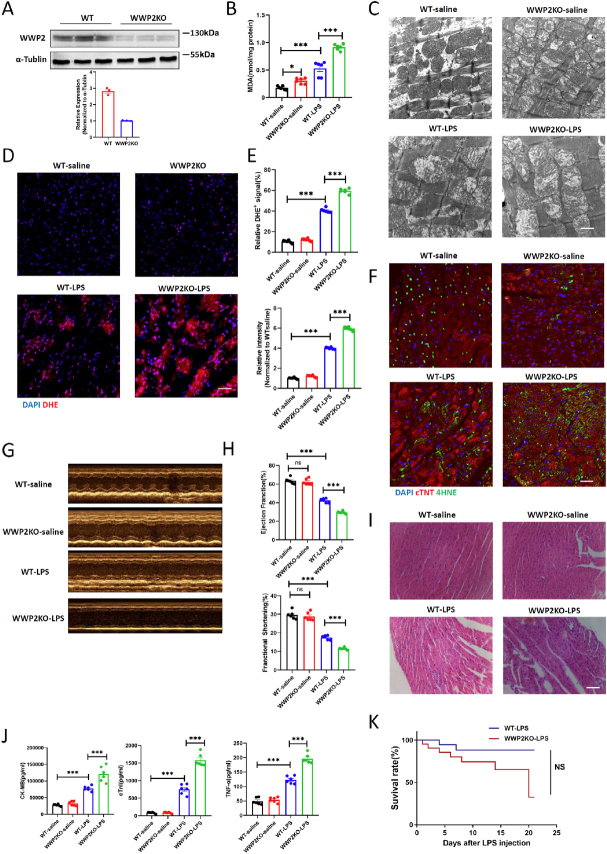
LPS-induced myocardial injury and ferroptosis is exacerbated in WWP2 knockout mice. (A) Western blotting of WWP2 in WT and WWP2 knockout (WWP2KO) mice (*N* = 3). (B) Determination of MDA levels in WT and WWP2KO mice treated with saline or LPS (*N* = 3). (C) Representative TEM images of mitochondrial morphology and function in WT and WWP2KO mice treated with saline or LPS. Scale bar: 800 nm. (D-E) Representative IF staining and quantification of DHE in WT and WWP2KO mice treated with saline or LPS. Scale bar: 75 μm (*N* = 6). (F) Representative IF staining and quantification of 4-HNE in WT and WWP2KO mice treated with saline or LPS. Scale bar: 75 μm (*N* = 6). (G-H) Representative images of echocardiogram and quantification of ejection fraction (EF) and fractional shortening (FS) in WT and WWP2KO mice treated with saline or LPS (*N* = 6). (I) Representative H& E staining of myocardium in WT and WWP2KO mice treated with saline or LPS. Scale bar: 50 μm. (J) Detection of plasma levels of CK-MB, cTnl and TNF-α in WWP2KO mice treated with saline or LPS (*N* = 6). (K) Survival rate of WT and WWP2KO mice after treated with saline or LPS. Data were presented as the mean±SEM. NS, not significant. ^*^P<0.05, ^***^P<0.001.

In order to rigorously elucidate the impact of WWP2 on LPS-induced myocardial injury and cardiomyocytes ferroptosis, we generated WWP2KO mice and confirmed the efficiency of knockout through Western blot (Figure 6A). Notably, the ubiquitination levels of FACL4 were also increased in WWP2KO mice after LPS treatment, which suggested enhanced modulation by WWP2 (Supplemental Figure 3). Subsequently, we assessed the levels of malondialdehyde (MDA) and mitochondrial morphology in the myocardium of the different groups. The results indicated that WWP2KO led to increased MDA levels in myocardial tissue and exacerbated the mitochondrial morphological changes induced by LPS injury (Figure 6B-C). DHE and 4HNE staining results revealed that WWP2KO exacerbated oxidative stress damage in the myocardium and promoted cardiomyocytes ferroptosis (Figure 6D-F). Using cardiac ultrasound examination, H& E staining, and serum injury marker analysis, we observed that WWP2KO mice displayed more pronounced myocardial damage and worsening of cardiac function following LPS treatment (Figure 6G-J, Supplemental Figure 4). Additionally, we monitored the survival of wild-type mice and WWP2KO mice after LPS treatment. The results demonstrated a moderate reduction in the survival rate of the WWP2KO group of mice (Figure 6K). Collectively, these findings underscore the significant role of WWP2 in modulating LPS-induced myocardial injury and cardiomyocytes ferroptosis, suggesting that WWP2 deficiency exacerbates the adverse effects associated with SICI.

### Knockdown of FACL4 Rescues the Adverse Effects of WWP2KO on Cardiac Injury and Cardiomyocytes Ferroptosis

**Figure 7:**
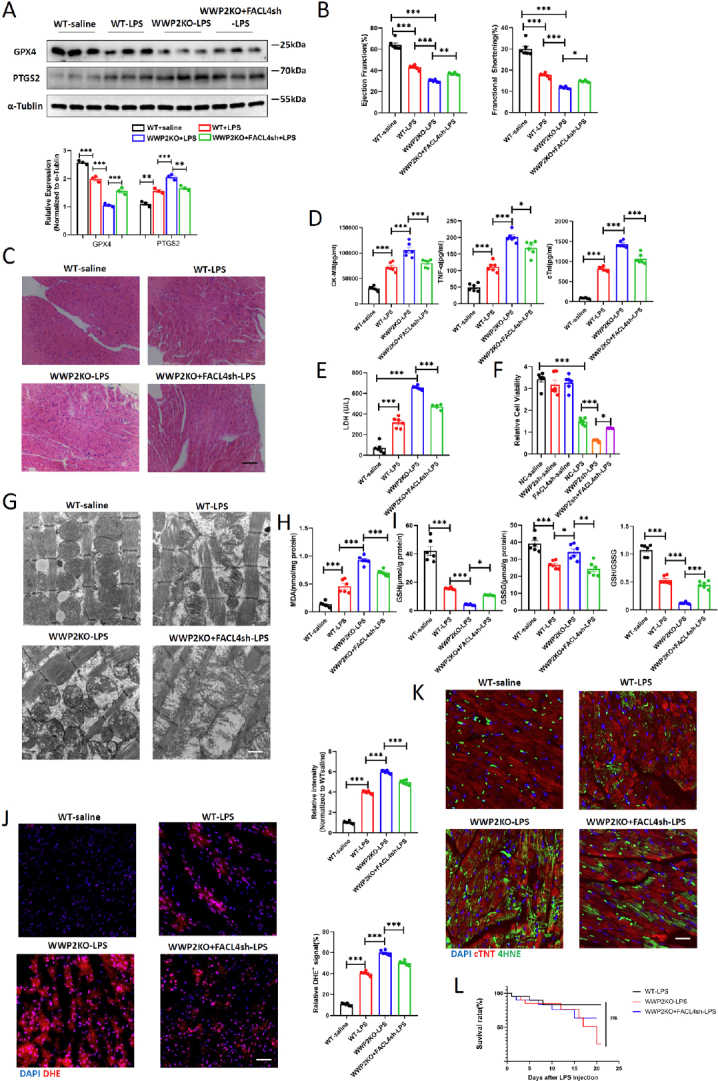
Knockdown of FACL4 rescues the adverse effects of WWP2 knockout on cardiac injury and cardiomyocytes ferroptosis. (A) Western blotting and quantification of GPX4 and PTGS2 in WT and WWP2 knockout (WWP2KO) mice injected with AAV9-CTNT-FACL4sh and treated with LPS (*N* = 3). (B) Quantification of EF and FS in WT and WWP2KO mice injected with AAV9-CTNT-FACL4sh and treated with LPS (*N* = 6). (C) Representative H& E staining of myocardium in injected with AAV9-CTNT-FACL4sh and treated with LPS. Scale bar: 50 μm. (D) Detection of plasma levels of CK-MB, cTnl and TNF-α in WT and WWP2KO mice injected with AAV9-CTNT-FACL4sh and treated with LPS (*N* = 6). (E) Detection of LDH levels in WT and WWP2KO mice injected with AAV9-CTNT-FACL4sh and treated with LPS (*N* = 6). (F) Cell viability quantified by CCK-8 assays (*N* = 6). (G) Representative TEM images of mitochondrial morphology and function in WT and WWP2KO mice injected with AAV9-CTNT-FACL4sh and treated with LPS. Scale bar: 800 nm. (H) Determination of MDA levels in WT and WWP2KO mice injected with AAV9-CTNT-FACL4sh and treated with LPS (*N* = 6). (I) Determination of glutathione (GSH), Oxidized glutathione (GSSG) and GSH/GSSG ratio in H9C2 cells (*N* = 6). (J) Representative IF staining and quantification of DHE in WT and WWP2KO mice injected with AAV9-CTNT-FACL4sh and treated with LPS. Scale bar: 75 μm (*N* = 6). (K) Representative IF staining and quantification of 4-HNE in WT and WWP2KO mice injected with AAV9-CTNT-FACL4sh and treated with LPS. Scale bar: 75 μm. (L) Survival rate of WT and WWP2KO mice injected with AAV9-CTNT-FACL4sh after treated with saline or LPS. Data were presented as the mean±SEM. ^*^P<0.05, ^**^P<0.01, ^***^P<0.001.

Next, we further validated the involvement of FACL4 in the regulation of WWP2-mediated LPS-induced myocardial injury and mitochondrial pathways associated with cardiomyocytes ferroptosis. To achieve this, we administered AAV9-CTNT-FACL4sh in WWP2KO mice to inhibit myocardial FACL4 expression. The western blot results showed that FACL4 knockdown alleviated ferroptosis induced by WWP2KO after LPS treatment (Figure 7A). The results from cardiac ultrasound, H& E staining, LDH detection and serum marker analysis indicated that suppressing FACL4 could rescue the exacerbated LPS-induced myocardial damage and cardiac functional decline observed in the WWP2KO group (Figure 7B-E, Supplemental Figure 5A-B). CCK-8 (cell counting kit-8) assay results suggested that inhibiting FACL4 could partially mitigate the negative impact on cell viability caused by WWP2 downregulation (Figure 7F). Transmission electron microscopy results indicated that suppressing FACL4 could ameliorate the mitochondrial morphological disruption caused by WWP2 deficiency (Figure 7G). Furthermore, the content of GSH in WWP2KO myocardial tissue significantly increased after downregulating FACL4, while the levels of MDA and GSSG markedly decreased (Figure 7H-I). Moreover, we found that inhibiting FACL4 significantly improved myocardial oxidative stress damage and ferroptosis levels in the sepsis model of WWP2KO mice (Figure 7J-K). Despite the survival rate was mildly increased by FACL4 knockdown, this difference was not statistically significant (Figure 7L). Therefore, our comprehensive findings strongly imply that WWP2 exerts its primary inhibitory influence on cardiomyocytes ferroptosis, thus protecting against LPS-induced myocardial injury. This regulatory cascade is mediated through the process of ubiquitination and degradation of FACL4.

## Discussion

WWP2 is a member of the HECT (homologous to the E6-AP carboxyl terminus)-type ligases NEDD4 (neuronally expressed developmentally downregulated 4) family, participating in various physiological activities including intracellular signaling, protein transport, and post-translational modifications.^[26]^ In the field of cardiovascular diseases, our previous studies have demonstrated that WWP2 can improve Angiotensin II - induced heart failure and ventricular remodeling by selectively ubiquitinating and degrading PARP1.^[27]^ Additionally, in vascular endothelial cells, we have revealed that WWP2 can alleviate oxidative stress and vascular endothelial damage induced by T2DM (type 2 diabetes mellitus) through downstream signaling molecules like Septin4 and DDX3X(dead-box helicase 3 x-linked).^[28,29]^ In this current study, our findings have unveiled WWP2 as a novel and pivotal regulator within the context of sepsis-induced myocardial injury. Across both *in vivo* and *in vitro* sepsis models, a consistent trend of decreased WWP2 protein expression was observed. Through the strategic utilization of cardiac-targeted WWP2 intervention vectors and WWP2KO mice, we have meticulously demonstrated WWP2’s capacity to mitigate LPS-induced myocardial injury. The mechanism underlying WWP2’s protective effects is illuminated by its role in ubiquitinating and subsequently degrading FACL4. This orchestrated process effectively curtails mitochondrial oxidative stress damage and curbs the occurrence of ferroptosis induced by LPS stimulation. This research provides new insights and intervention targets for understanding the pathogenesis and treatment of sepsis-induced cardiac injury.

Sepsis represents an acute syndrome with far-reaching impacts on multiple organ systems. Within this landscape, myocardial injury triggered by sepsis stands as a primary contributor to both mortality and disability. However, the precise underlying mechanisms driving SICI remain a complex puzzle awaiting full elucidation. Within this context, emerging research highlights several potential mechanisms, including acute systemic inflammatory responses, cascades of cytokines, programmed cell death, oxidative stress, and perturbations in mitochondrial function.^[30]^ Inflammation-linked myocardial damage takes center stage in infectious myocarditis, manifesting through the release of inflammatory factors that exhibit cardiac toxicity. Further complicating matters, the activation of the sympathetic nervous system leads to catecholamine toxicity.^[31,32]^ Our research endeavors delved into the intricate interplay. In the presence of LPS treatment, significant elevations in serum TNF levels and the expression of myocardial damage markers like cTNI and CK-MB were observed. Remarkably, the overexpression of WWP2 acted as a mitigating force, substantially attenuating the elevation of these indicators of injury. From the perspective of cardiac morphology and function, SICI resulted in substantial inflammatory infiltration, myocardial fiber dissolution, and further impairment of cardiac contractile function. On the other hand, several research indicate that LPS impairs cellular mitochondrial oxidative respiratory chain function, leading to the release of a large amount of ROS.^[33,34]^ In our WWP2KO mouse model of SICI, LPS-induced myocardial inflammatory responses and mitochondrial oxidative stress damage were aggravated. Conversely, through cardiac-specific overexpression of WWP2, we discovered that it stabilized the morphology and function of cardiomyocyte mitochondria, alleviated LPS-induced oxidative stress damage in the myocardium, maintained intracellular GSH redox homeostasis, and further improved the treatment of cardiac contractile dysfunction.

Programmed cell death (PCD) is a complex and precise form of cell death that plays a significant role in the development of various cardiovascular diseases.^[35]^ Aberrant activation of PCD can exacerbate myocardial injury and ventricular remodeling, making proper inhibition of PCD a potential effective approach for treating various cardiovascular disorders.^[36]^ Previous studies have indicated that apoptosis and ferroptosis are closely associated with the pathogenesis of SICI.^[37]^ However, it remains unknown which type of PCD plays a more crucial role in the process of SICI. In our research, we found that compared to the apoptosis inhibitor z-VAD-FMK, the ferroptosis inhibitor Fer-1 could more effectively alleviate the myocardial damage induced by LPS. Moreover, although WWP2 can to some extent suppress LPS-induced myocardial cell apoptosis, its main impact is on ferroptosis. In both *in vivo* and *in vitro* SICI models, inhibiting WWP2 led to varying degrees of increased cardiomyocytes apoptosis and ferroptosis. In comparison to z-VAD-FMK, the use of Fer-1 significantly improved cardiomyocytes survival, alleviated impaired cardiac contractile function, and reduced myocardial damage. Therefore, the cardioprotective effect of WWP2 is largely achieved through inhibiting the mechanism of ferroptosis in cardiomyocytes. This intricate interplay among WWP2, ferroptosis, and the landscape of SICI holds clinical significance. While animal and cell models inevitably diverge from the complex human scenario of sepsis, our findings offer a translational foundation for harnessing ferroptosis inhibition in the pursuit of treating myocardial damage caused by sepsis. This has particular resonance for clinical SICI patients who currently face a dearth of specific and effective treatments, as our research introduces fresh perspectives on potential therapeutic avenues. In our study, we supplemented the understanding of the relationship between myocardial inflammation and ferroptosis in the SICI process from different perspectives. We explained the specific mechanism of ferroptosis occurrence in the context of SICI from the angles of myocardial inflammation and mitochondrial oxidative stress responses. The role of mitochondria in the mechanism of ferroptosis has been previously reported. Systemic inflammation triggered by LPS may disrupt the oxidative respiratory function homeostasis of mitochondria within cardiomyocytes, resulting in an imbalance of ROS.^[38]^ Our study discovered that after WWP2 intervention, changes in mitochondrial morphology and lipid peroxidation in cardiomyocytes corresponded to the typical features of ferroptosis. Together with previous research, these pieces of evidence provide a more detailed exploration of the predominant type of PCD, namely ferroptosis, in the context of SICI. In summary, our research results offer a more comprehensive understanding of cardiomyocytes ferroptosis in the SICI process, and shed light on further discussions about employing ferroptosis as the primary PCD type for treating SICI.

An increasing body of research indicates that FACL4 is one of the key proteins involved in regulating cell ferroptosis.^[39,40]^ In this process, FACL4 primarily esterifies CoA into free fatty acids in an adenosine triphosphatedependent manner. Furthermore, ASCL4 (Acyl-CoA synthetase long-chain family member 4) shows a distinct preference for long polyunsaturated fatty acids, such as AA and adrenoyl acid (AdA). This study has identified FACL4 as a downstream target through which WWP2 regulates both physiological processes and the pathogenesis of SICI in cardiomyocytes ferroptosis. WWP2 is involved in the metabolic pathway of PUFAs through FACL4 to maintain the stability of mitochondrial membranes in myocardial cells, thereby reducing intracellular lipid ROS. Specifically, WWP2 directly binds to FACL4, promoting its ubiquitination and subsequent degradation through the proteasomal pathway, thus inhibiting myocardial cell ferroptosis and ameliorating SICI. Moreover, our findings indicate that through the downregulation of FACL4, the detrimental effects on myocardial injury and cardiac function caused by WWP2KO can be rescued in transgenic mouse models. Our research reveals a novel ubiquitination pathway that regulates FACL4 protein levels within cardiomyocytes. Simultaneously, it provides deeper insights into the mechanisms of ubiquitination modification and the pathogenesis of SICI.

This study has several limitations. First, despite we have demonstrated that WWP2 directly bound with and promoted the ubiquitination dependent degradation of FACL4, further investigations are required to provide an in-depth mechanistic description on the biological role of WWP2-FACL4 axis in sepsis-induced cardiac injury. Second, while we utilized myocardium-specific WWP2 intervention vectors to investigate the impact of WWP2 on LPS-induced myocardial injury and ferroptosis, we have not explored the functional and mechanistic aspects of systemic sepsis-related damage in other organs in WWP2KO mice. Third, the translational value of WWP2 as an application in the evaluation and treatment of infectious cardiomyopathy needs to be further verified in preclinical and clinical studies in the future. Forth, despite having provided reliable evidence showing the impact of WWP2 on LPS-induced ferroptosis in cardiomyocytes, we failed to provide comprehensive detection of ferroptosis related changes including intracellular ion content. These aspects need to be further elucidated in our future research endeavors.

In conclusion, this study conducted *in vitro* and *in vivo* experiments to investigate how WWP2 regulates oxidative stress imbalance and ferroptosis in the context of sepsis-induced cardiac injury. Through the ubiquitination-mediated degradation of FACL4, WWP2 effectively inhibits oxidative stress and ferroptosis in cardiomyocytes, ultimately leading to the improvement of myocardial damage and restoration of cardiac function. These findings underscore the significant role of WWP2 in the processes of SICI and cardiomyocytes ferroptosis, suggesting that WWP2 could potentially serve as a promising target for clinical interventions aiming to mitigate heart damage induced by sepsis.

## Supplementary Material

Supplementary Material

## References

[1] Gotts JE, Matthay MA (2016). Sepsis: pathophysiology and clinical management. BMJ.

[2] Liu Y, Liu Y, Ye S, Feng H, Ma L (2023). A new ferroptosis-related signature model including messenger RNAs and long non-coding RNAs predicts the prognosis of gastric cancer patients. J Transl Int Med.

[3] Vincent JL (2022). Current sepsis therapeutics. EBioMedicine.

[4] Pant A, Mackraj I, Govender T (2021). Advances in sepsis diagnosis and management: a paradigm shift towards nanotechnology. J Biomed Sci.

[5] Lv X, Wang H (2016). Pathophysiology of sepsis-induced myocardial dysfunction. Mil Med Res.

[6] Hollenberg SM, Singer M (2021). Pathophysiology of sepsis-induced cardio-myopathy. Nat Rev Cardiol.

[7] Merx MW, Weber C (2007). Sepsis and the heart. Circulation..

[8] Yang H, Zhang Z (2021). Sepsis-induced myocardial dysfunction: the role of mitochondrial dysfunction. Inflamm Res.

[9] Jiang X, Stockwell BR, Conrad M (2021). Ferroptosis: mechanisms, biology and role in disease. Nat Rev Mol Cell Biol.

[10] Tang D, Chen X, Kang R, Kroemer G (2021). Ferroptosis: molecular mechanisms and health implications. Cell Res.

[11] Stockwell BR, Jiang X, Gu W (2020). Emerging Mechanisms and Disease Relevance of Ferroptosis. Trends Cell Biol.

[12] Fang X, Wang H, Han D, Xie E, Yang X, Wei J (2019). Ferroptosis as a target for protection against cardiomyopathy. Proc Natl Acad Sci U S A.

[13] Tadokoro T, Ikeda M, Ide T, Deguchi H, Ikeda S, Okabe K (2023). Mitochondria-dependent ferroptosis plays a pivotal role in doxorubicin cardiotoxicity. JCI Insight.

[14] Li N, Wang W, Zhou H, Wu Q, Duan M, Liu C (2020). Ferritinophagy-mediated ferroptosis is involved in sepsis-induced cardiac injury. Free Radic Biol Med.

[15] Pohl C, Dikic I (2019). Cellular quality control by the ubiquitin-proteasome system and autophagy. Science.

[16] Kodron A, Mussulini BH, Pilecka I, Chacińska A (2021). ’The ubiquitin-protea-some system and its crosstalk with mitochondria as therapeutic targets in medicine. Pharmacol Res.

[17] Li H, Zhang Z, Wang B, Zhang J, Zhao Y, Jin Y (2007). Wwp2-mediated ubiq-uitination of the RNA polymerase II large subunit in mouse embryonic pluripotent stem cells. Mol Cell Biol.

[18] Maddika S, Kavela S, Rani N, Palicharla VR, Pokorny JL, Sarkaria JN (2011). WWP2 is an E3 ubiquitin ligase for PTEN. Nat Cell Biol.

[19] Chen W, Jiang X, Luo Z (2014). WWP2: a multifunctional ubiquitin ligase gene. Pathol Oncol Res.

[20] Xu H, Wang W, Li C, Yu H, Yang A, Wang B (2009). WWP2 promotes degradation of transcription factor OCT4 in human embryonic stem cells. Cell Res.

[21] Mokuda S, Nakamichi R, Matsuzaki T, Ito Y, Sato T, Miyata K (2019). Wwp2 maintains cartilage homeostasis through regulation of Adamts5. Nat Commun.

[22] Zhou Y, Zhou H, Hua L, Hou C, Jia Q, Chen J (2021). Verification of ferroptosis and pyroptosis and identification of PTGS2 as the hub gene in human coronary artery atherosclerosis. Free Radic Biol Med.

[23] Gan B (2021). Mitochondrial regulation of ferroptosis. J Cell Biol.

[24] Ta N, Qu C, Wu H, Zhang D, Sun T, Li Y (2022). Mitochondrial outer membrane protein FUNDC2 promotes ferroptosis and contributes to doxorubicin-induced cardiomyopathy. Proc Natl Acad Sci U S A.

[25] Sung YK, Park MK, Hong SH, Hwang SY, Kwack MH, Kim JC (2007). Regulation of cell growth by fatty acid-CoA ligase 4 in human hepatocellular carcinoma cells. Exp Mol Med.

[26] Zou W, Chen X, Shim JH, Huang Z, Brady N, Hu D (2011). The E3 ubiquitin ligase Wwp2 regulates craniofacial development through mono-ubiquitylation of Goosecoid. Nat Cell Biol.

[27] Zhang N, Zhang Y, Qian H, Wu S, Cao L, Sun Y (2020). Selective targeting of ubiquitination and degradation of PARP1 by E3 ubiquitin ligase WWP2 regulates isoproterenol-induced cardiac remodeling. Cell Death Differ.

[28] Zhang N, Zhang Y, Wu B, You S, Sun Y (2020). Role of WW domain E3 ubiquitin protein ligase 2 in modulating ubiquitination and Degradation of Septin4 in oxidative stress endothelial injury. Redox Biol.

[29] You S, Xu J, Yin Z, Wu B, Wang P, Hao M (2023). Down-regulation of WWP2 aggravates Type 2 diabetes mellitus-induced vascular endothelial injury through modulating ubiquitination and degradation of DDX3X. Cardiovasc Diabetol.

[30] Carbone F, Liberale L, Preda A, Schindler TH, Montecucco F (2022). Septic Cardiomyopathy: From Pathophysiology to the Clinical Setting. Cells.

[31] Beesley SJ, Weber G, Sarge T, Nikravan S, Grissom CK, Lanspa MJ (2018). Septic Cardiomyopathy. Crit Care Med.

[32] Ehrman RR, Sullivan AN, Favot MJ, Sherwin RL, Reynolds CA, Abidov A (2018). Pathophysiology, echocardiographic evaluation, biomarker findings, and prognostic implications of septic cardiomyopathy: a review of the literature. Crit Care.

[33] Mills EL, Kelly B, Logan A, Costa ASH, Varma M, Bryant CE (2016). Succinate Dehydrogenase Supports Metabolic Repurposing of Mitochondria to Drive Inflammatory Macrophages. Cell.

[34] Jha AK, Huang SC, Sergushichev A, Lampropoulou V, Ivanova Y, Loginicheva E (2015). Network integration of parallel metabolic and transcriptional data reveals metabolic modules that regulate macrophage polarization. Immunity.

[35] Li M, Wang ZW, Fang LJ, Cheng SQ, Wang X, Liu NF (2022). Programmed cell death in atherosclerosis and vascular calcification. Cell Death Dis.

[36] Zhou L, Sun J, Gu L, Wang S, Yang T, Wei T (2021). Programmed Cell Death: Complex Regulatory Networks in Cardiovascular Disease. Front Cell Dev Biol.

[37] Yang Z, Pan X, Wu X, Lin Q, Chen Y, Cai S (2023). TREM-1 induces pyroptosis in cardiomyocytes by activating NLRP3 inflammasome through the SMC4/NEMO pathway. FEBS J.

[38] Li Y, Feng YF, Liu XT, Li YC, Zhu HM, Sun MR (2021). Songorine promotes cardiac mitochondrial biogenesis via Nrf2 induction during sepsis. Redox Biol.

[39] Ji Q, Fu S, Zuo H, Huang Y, Chu L, Zhu Y (2022). ACSL4 is essential for radiation-induced intestinal injury by initiating ferroptosis. Cell Death Discov.

[40] Sun Z, Wu J, Bi Q, Wang W (2022). Exosomal lncRNA TUG1 derived from human urine-derived stem cells attenuates renal ischemia/reperfusion injury by interacting with SRSF1 to regulate ASCL4-mediated ferroptosis. Stem Cell Res Ther.

