# Erratum to "WWP2 protects against sepsis-induced cardiac injury through inhibiting cardiomyocyte ferroptosis"

**DOI:** 10.1515/jtim-2024-0024

**Published:** 2024-11-06

**Authors:** Zhi Li, Boquan Wu, Jie Chen, Ning Ye, Rui Ma, Chunyu Song, Yingxian Sun, Xingang Zhang, Guozhe Sun

**Affiliations:** Department of Cardiology, The First Hospital of China Medical University, Shenyang 110001, Liaoning Province, China

## Connected Content

This corrects the article "WWP2 protects against sepsis-induced cardiac injury through inhibiting cardiomyocyte ferroptosis" published in 2024 [J Transl Int Med. 2024; 12(1): 35-50. DOI: 10.2478/jtim-2024-0004].

In the published article, Figure 1A was mistakenly selected when the authors composed the manuscript. Although this error does not impact the core findings or conclusions of the study, the authors wish to update the Figure 1 in this Erratum and amend the relevant content to ensure the quality of the article. Erratum to the Figure 1 is presented below.

**Erratum to Figure 1:**
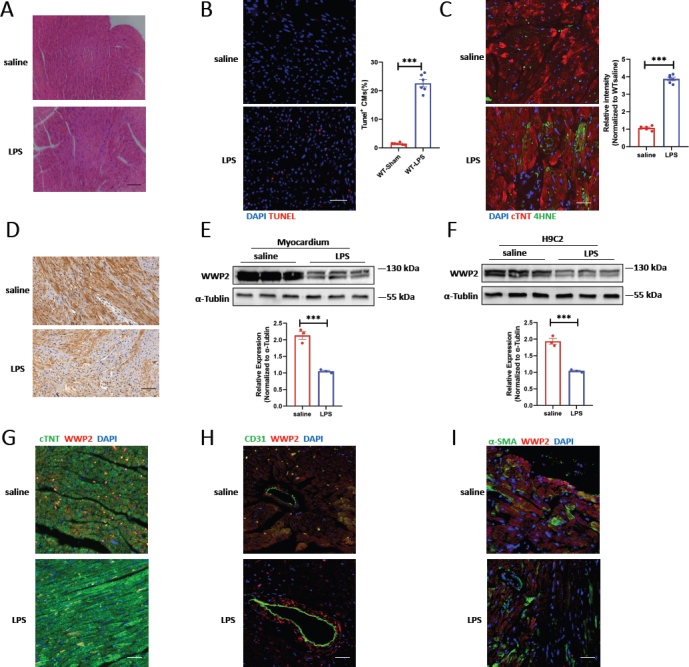
WWP2 is downregulated in septic heart and cardiomyocytes. A. Representative H&E staining of myocardium in adult mice treated with saline or LPS. Scale bar: 50 μm. B. Representative immunofluorescence (IF) staining and quantification of Tunel^+^ cardiomyocytes in myocardium treated with saline or LPS. Scale bar: 100 μm (*N* = 6). C. Representative immunofluorescence staining and quantification of 4-HNE in myocardium treated with saline or LPS. Scale bar: 75 μm (*N* = 6). D. Representative immunohistochemistry (IHC) staining of WWP2 in myocardium treated with saline or LPS. Scale bar: 75 μm. E. Western blotting and quantification of WWP2 in in adult mice treated with saline or LPS (*N* = 3). F. Western blotting and quantification of WWP2 in in H9C2 cell line treated with saline or LPS (*N* = 3). G. Representative immunofluorescence (IF) staining of WWP2 and cardiomyocytes (cTNT) in myocardium treated with saline or LPS. Scale bar: 50 μm. H. Representative immunofluorescence (IF) staining of WWP2 and Endothelial cells (CD31) in myocardium treated with saline or LPS. Scale bar: 50 μm. I. Representative immunofluorescence (IF) staining of WWP2 and fibroblasts (α-SMA) in myocardium treated with saline or LPS. Scale bar: 50 μm. Data were presented as the mean±SEM. ***P<0.001.

