# Supplementary material for: WWP2 protects against sepsis-induced cardiac injury through inhibiting cardiomyocyte ferroptosis
Source: J Transl Int Med. 2024 Mar 21;12(1):35–50. doi: 10.2478/jtim-2024-0004 (PMC11000860; doi:10.2478/jtim-2024-0004)
Supplement: Supplementary file 1 — Supplementary Material [file jtim-2024-0004_sm.pdf]

Supplementary Methods

Adeno-associated virus 9 (AAV9), plasmids and shRNA construction

For *in vivo* study, AAV9-cTNT-Flag-WWP2 was used to increase WWP2 expression in cardiomyocytes and AAV9-cTNT-Flag-CON was used as control. AAV9- cTNT-WWP2sh or AAV9- cTNT-FACL4sh were used to knockdown WWP2 or FACL4 respectively. The plasmid sequence for WWP2 overexpression was: 5'-
ATGGCATCTGCCAGCTCCAGCCGGGCAGGAGTGGCCCTGCCTTTTGAGAAGTCCCAGCTTACCC
TGAAAGTGGTGTCAGCAAAGCCCAAGGTGCACAACCGCCAGCCCAGAATCAACTCCTACGTGG
AGGTGGCAGTGGATGGACTCCCCAGCGAGACCAAGAAGACGGGGAAGCGCATCGGGAGCTCT
GAACTGCTCTGGAATGAAATCATCGTTCTGAATGTCACAGCCCAGAGTCATTTGGATCTGAAG
GTCTGGAGCTGTCATACCTTGAGGAATGAACTACTGGGCACTGCCTCTGTCAACCTCTCCAATG
TCCTGAAGAACAATGGCGGCAAAATGGAGAACACACAACTGACCCTGAACCTGCAGACAGAG
AACAAAGGCAGTGTTGTCTCGGGAGGAGAGCTGACAATTTTCCTGGATGGGCCAACTGTTGAT
CTGGGAAGTGTGCCTAATGGCAGTGCAGTGACAGACGGATCACAGCCACCTTCAAGAGAATCC
AGTGGGACTGCTATAGCTCCAGAGACCCGGCACCAGCCCCCCAGTACAAACTGCTTTGGTGGC
AGATCCCGGACGCACAGACACTCAGGTGGCTCAGCCAGGACAGCCACAGCAGCCAGTGAACA
AAGCCCTGGCGCTAGGAACCGCCACCGCCAGCCTGTGAAGAACTCTAGCAGCAGTGGCTTAGC
CAATGGCACAGTGAATGAGGAACCTACTCCAGCCAGTGAACCTGAAGAATCGTCGGTTGTTGG
TGTAACATCCCTGCCTGCAGCAGCCTTGAGTGTGTCCTCAAATCCCAACACAACATCTCTCCCT
GCACAGTCCACACCAGCAGAGGGAGAGGAGGCCAGCACTTCCGGGACACAGCAGCTCCCTGC
TGCCGCCCAGGCCCCTGATGCTCTTCCTGCTGGATGGGAACAGAGAGAGCTGCCCAATGGGCG
TGTCTATTATGTTGATCACAACACCAAGACCACCACCTGGGAGCGGCCTCTTCCTCCAGGGTGG
GAAAAGCGCACGGACCCCCGAGGGAGGTTTTACTACGTGGACCACAACACCCGGACAACCAC
CTGGCAGCGCCCAACTGCTGAGTACGTGCGCAACTATGAGCAGTGGCAGTCCCAGCGGAACCA
GCTGCAGGGGGCCATGCAGCACTTCAGCCAAAGATTCCTCTACCAGTCTTCGAGTGCTTCGACT
GACCATGATCCCTTGGGCCCCCTCCCACCTGGCTGGGAGAAGAGGCAGGACAATGGACGGGTG
TATTATGTCAACCACAACACTCGCACTACCCAGTGGGAGGACCCTCGGACCCAGGGGATGATA
CAGGAGCCAGCCCTGCCCCCAGGGTGGGAGATGAAATACACCAGCGAGGGCGTGCGGTACTTT
GTGGACCACAATACCCGCACCACTACCTTTAAGGATCCTCGCCCAGGGTTCGAGTCAGGGACA
AAGCAAGGCTCACCTGGTGCCTATGACCGAAGTTTTCGGTGGAAGTATCACCAGTTCCGTTTCC
TCTGCCACTCAAATGCTCTACCCAGCCATGTGAAGATCAGCGTTTCCAGGCAGACACTCTTTGA
GGATTCTTTCCAACAGATTATGAACATGAAACCTTACGACCTGCGCCGCCGGCTCTACATCATC
ATGCGTGGTGAGGAGGGCCTGGACTACGGCGGCATCGCCAGAGAGTGGTTTTTCCTCCTGTCCCATGA
GGTGCTCAACCCTATGTACTGTTTGTTTGAATATGCTGGGAAGAACAATTACTGCCTGC
AGATCAACCCGGCCTCTTCCATCAACCCTGACCACCTCACCTACTTCCGCTTTATCGGCAGATT

CATCGCCATGGCTCTGTACCATGGGAAGTTCATCGACACAGGCTTCACTCTCCCTTTCTACAAG
CGGATGCTCAACAAGAGACCGACTCTGAAGGACCTGGAGTCTATTGACCCTGAGTTTTACAAC
TCCATTGTCTGGATCAAAGAGAACAACCTGGAAGAGTGTGGTCTGGAGCTGTTTTTCATCCAGG
ACATGGAGATTCTGGGCAAGGTGACAACCCATGAACTGAAGGAGGGCGGTGAGAACATCCGA
GTTACCGAGGAGAACAAGGAGGAGTATATCATGCTGCTGACTGACTGGCGATTCACCCGAGGC
GTGGAAGAGCAGACCAAAGCTTTCCTGGATGGCTTCAATGAGGTTGCCCCTCTGGAGTGGTTG
AGATATTTTGATGAGAAAGAGCTGGAGCTCATGCTCTGCGGCATGCAGGAGATAGACATGAGC
GACTGGCAGAAGAACGCCATCTATCGGCACTACACCAAGAGCAGCAAGCAGATCCAGTGGTTC
TGGCAGGTTGTCAAGGAGATGGACAATGAGAAGAGGATCCGGCTACTGCAGTTTGTCACGGGA
ACCTGCCGTCTGCCTGTTGGGGGATTTGCTGAGCTCATCGGGAGCAATGGCCCGCAGAAGTTCT
GCATCGACAGAGTTGGCAAGGAAACCTGGCTGCCCAGGAGCCATACGTGCTTCAACCGTCTGG
ACCTGCCTCCCTATAAGAGCTACGAGCAGCTGAAAGAGAAGCTGCTGTACGCCATCGAGGAGA
CTGAGGGGTTCGGACAGGAGGGAGGTGGAGGATCAGACTACAAGGATGACGATGACAAGGAT
TACAAAGACGACGATGATAAGGACTATAAGGATGATGACGACAAATAG-3’. The target sequence of shRNA for WWP2 was 5’- TGGACAATGAGAAGAGGAT-3’. The target sequence of shRNA for FACL4 was 5’-ACAGCATGCAATCAGTAGA-3’.

For *in vitro* study, a HA-tagged full-length WWP2 plasmid was used to overexpress WWP2. WWP2 (WWP2 siRNA: GATCTGGGAAATGTGCCTA) and FALC4 (FACL4 siRNA: GAGCGATTTGAAATTCCAA) small interfering RNAs (siRNAs) were used to knockdown WWP2 and FACL4.

AAV9 vectors used in this study were purchased from Genechem (Shanghai, China). Small interfering RNAs were purchased from RIBO BIO (Guangzhou, China).

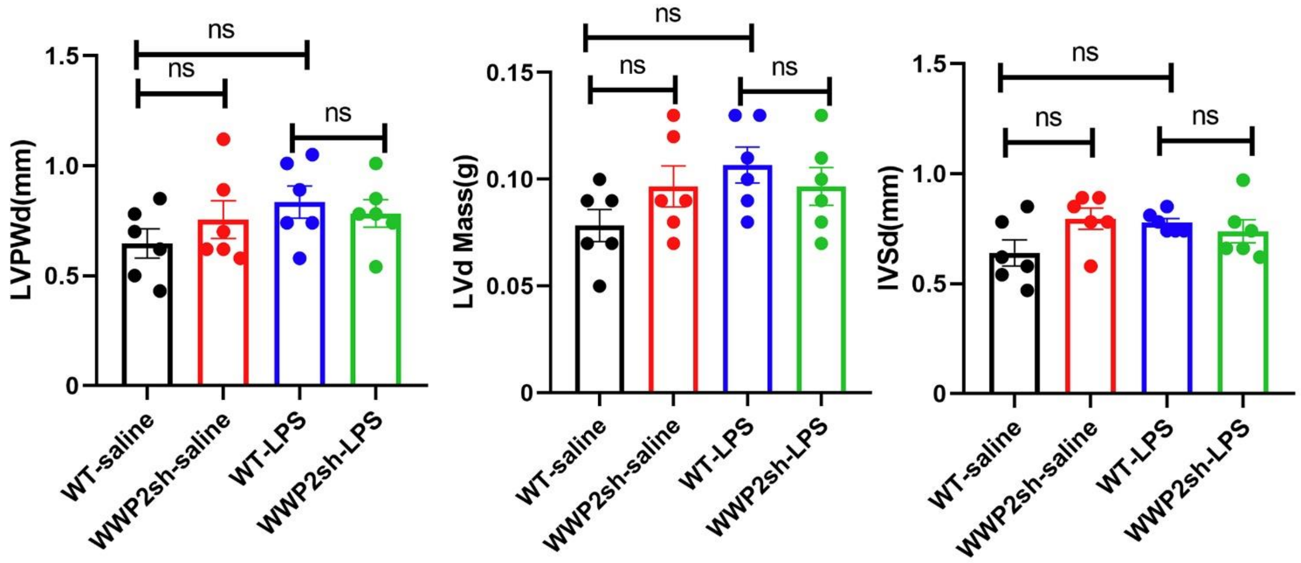

Supplementary Figure 1: Quantification of left ventricular posterior wall dimensions (LVPWd), interventricular septal thickness at diastole (IVSd) and left ventricular end diastolic (LVd) Mass from mice in Figure 2. Data were presented as the mean±SEM. NS, not significant (*N* = 6).

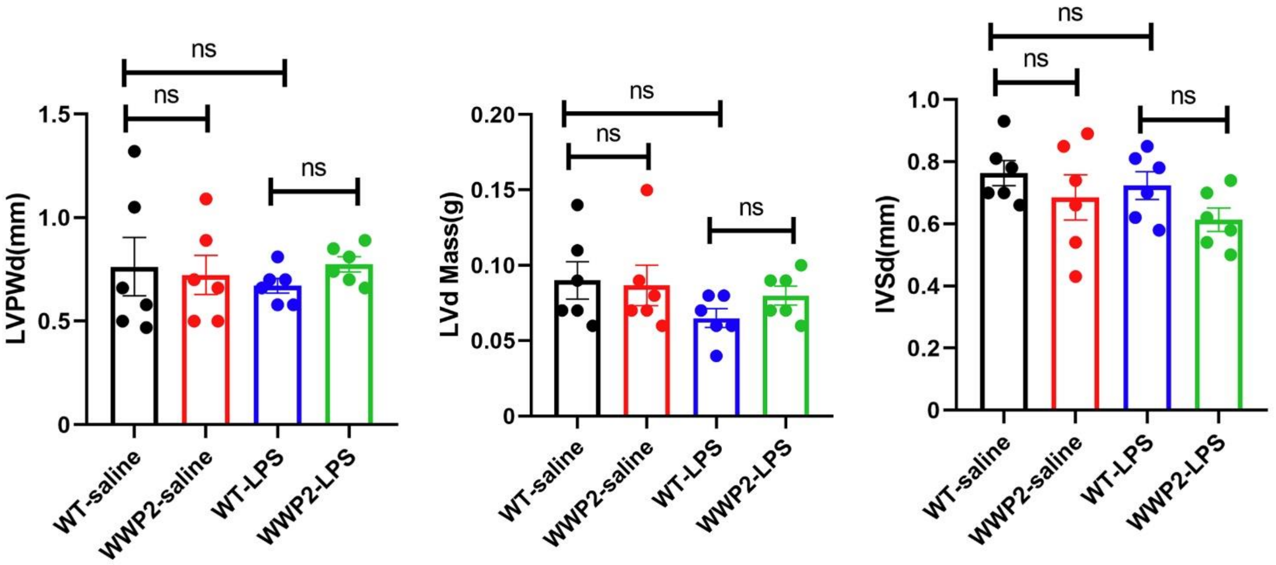

Supplementary Figure 2: Quantification of left ventricular posterior wall dimensions (LVPWd), interventricular septal thickness at diastole (IVSd) and left ventricular end diastolic (LVd) Mass from mice in Figure 3. Data were presented as the mean±SEM. NS, not significant (*N* = 6).

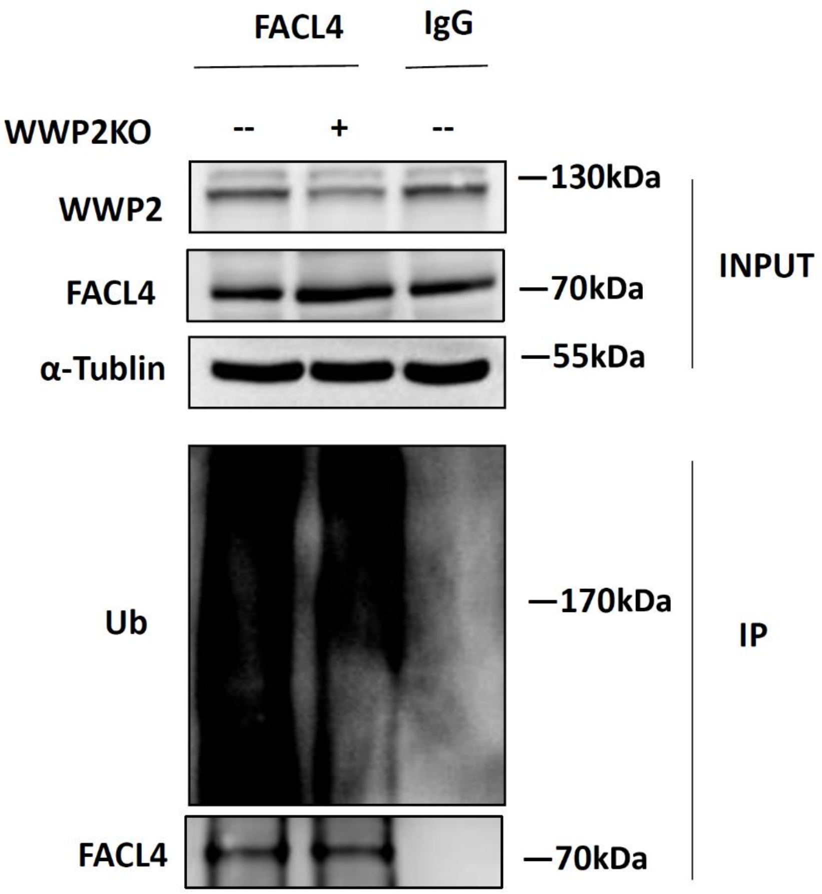

Supplementary Figure 3: Detection of ubiquitination levels in wild type (WT) and WWP2 knockout (WWP2KO) mice.

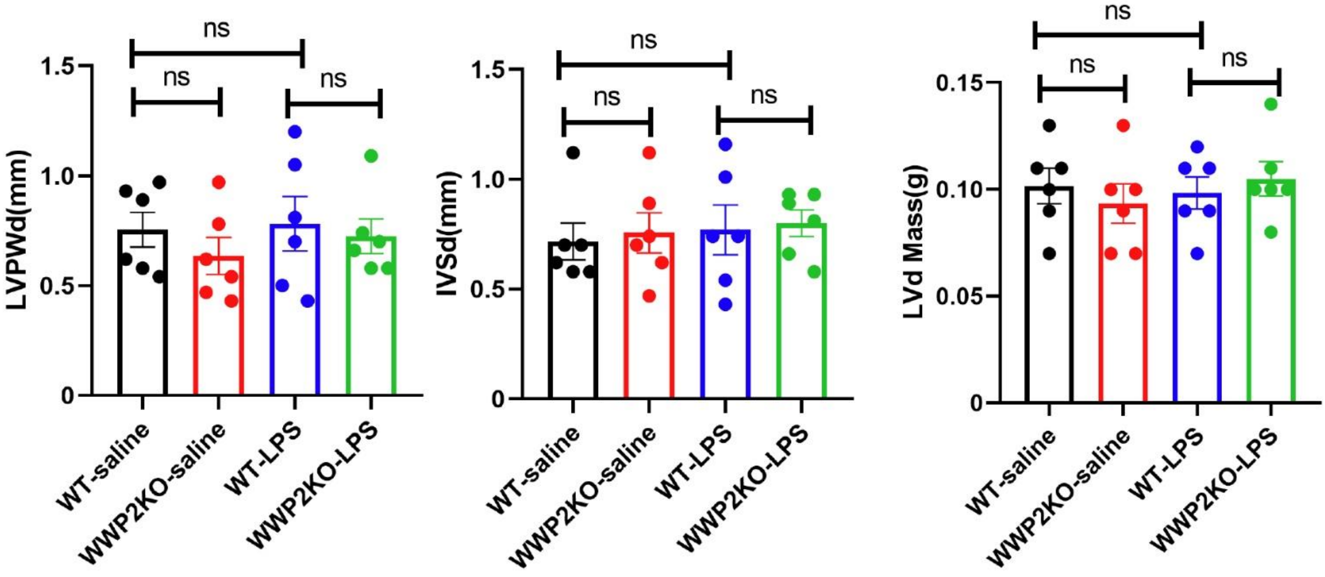

Supplementary Figure 4: Quantification of left ventricular posterior wall dimensions (VPWd), interventricular septal thickness at diastole (IVSd) and left ventricular end diastolic (LVd) Mass from mice in Figure 6. Data were presented as the mean±SEM. NS, not significant (*N* = 6).

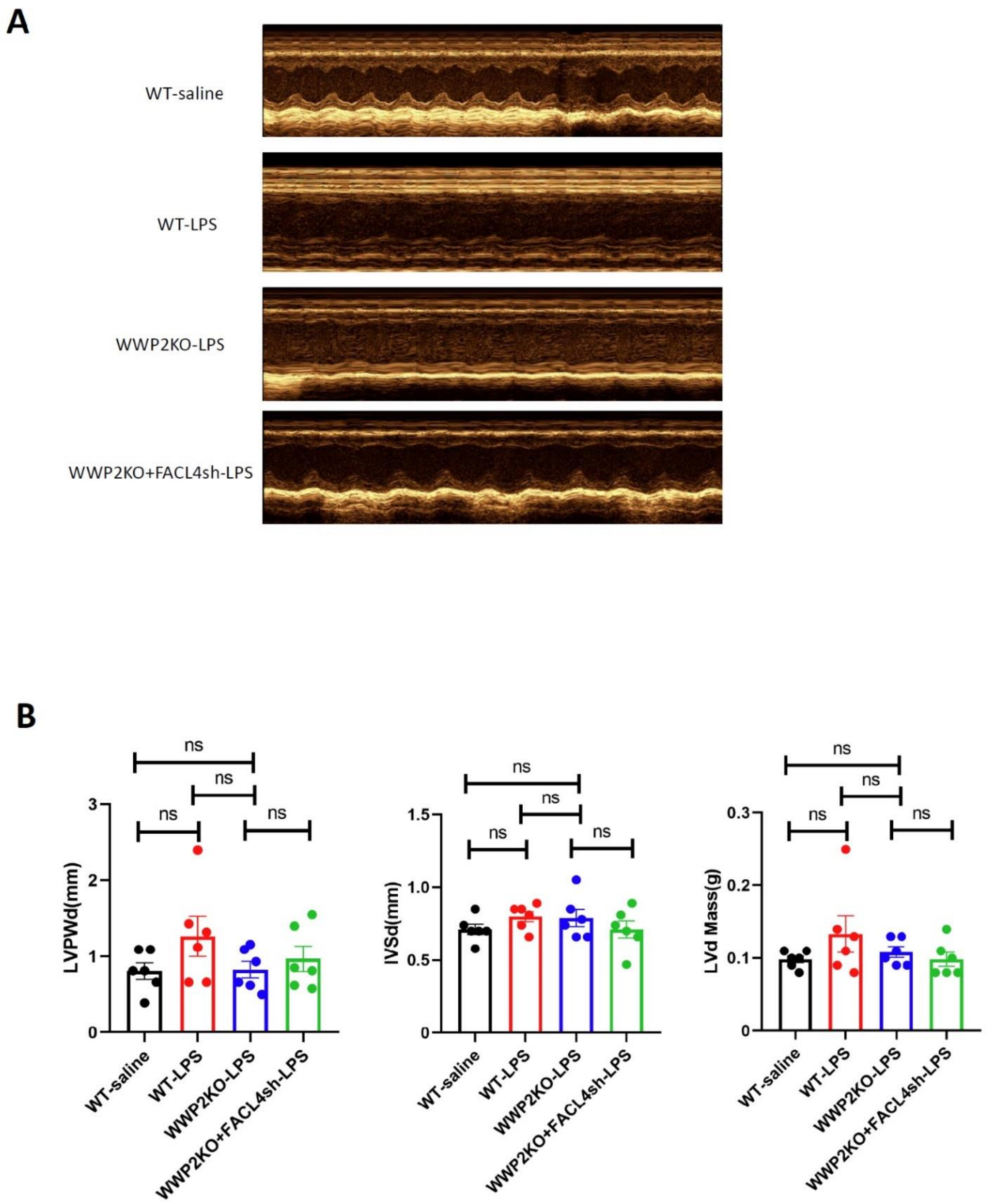

Supplementary Figure 5: Representative images and quantification of echocardiographic data in Figure 7. (A) Representative echocardiographic images in Figure 7. (B) Quantification of left ventricular posterior wall dimensions (LVPWd), interventricular septal thickness at diastole (IVSd) and left ventricular end diastolic (LVd) Mass from mice in Figure 7. Data were presented as the mean±SEM. NS, not significant (*N* = 6).
